# Correlation between salivary cytokine profiles and white spot lesions in adolescent patients receiving clear aligner orthodontic treatment

**DOI:** 10.1186/s12903-023-03561-3

**Published:** 2023-11-13

**Authors:** Qian Liu, Tao Guo, Wei Dang, Zhixin Song, Yi Wen, Houzhuo Luo, Axian Wang

**Affiliations:** 1 State Key Laboratory of Military Stomatology and National Clinical Research Center for Oral Diseases and Shaanxi Clinical Research Center for Oral Diseases, Department of Orthodontics, School of Stomatology, Air Force Medical University, Xi’an, China; 2 Department of Orthodontics, TaiKang Shanghai Bybo Dental Hospital, Shanghai, China; 3 Shaanxi Provincial Key Laboratory of Craniomaxillofacial Precision Medicine Research, Department of Prosthodontics, Stomatological Hospital of Xi’an Jiaotong University, Xi’an, 710004 China

**Keywords:** Invisible orthodontic correction, Adolescents, White spot lesion, Inflammatory cytokines

## Abstract

**Background:**

To explore the relationship between changes in salivary cytokine levels and the occurrence of white spot lesions in adolescents receiving clear aligner orthodontic treatment and investigate the predictive value of various factors for lesion occurrence.

**Methods:**

We retrospectively analyzed sixthy eight adolescent in the permanent dentition period, who received clear aligner orthodontics in our hospital were randomly divided into two groups according to the occurrence or aggravation of white spot lesions after treatment. The general condition of the oral cavity was analyzed, saliva was collected, and inflammation-related cytokines with varying transcription levels between groups were screened by transcriptome analysis. The expression levels of inflammatory cytokines in the saliva of the patients in the two groups were measured, and the risk factors for white spot lesions were screened by correlation analysis and binary logistic regression analysis. The value of the independent and combined application of risk factors for predicting the occurrence of white spot lesions in adolescent patients after invisible orthodontic treatment was analyzed by receiver operating characteristic (ROC) curve analysis.

**Results:**

Transcriptome and GO and KEGG pathway analyses showed that there were differences in the transcription levels of inflammatory cytokines such as CXCL1, CXCL2, CXCL8, CCL3, CCL4, IL-1β and IL-2 between groups. The levels of CXCL8, CCL3, CCL4, IL-1β and IL-2 in the saliva of patients with white spot lesions were significantly higher in patients after invisible orthodontic treatment (*P* < 0.05). Correlation analysis and binary logistic regression analysis showed that elevated levels of CXCL8, IL-1β and IL-2 were independent risk factors for the occurrence of white spot lesions (*P* < 0.05). CXCL8 had the highest independent predictive value for the occurrence of white spot lesions (AUC = 0.773, *P* < 0.05), and the combination of IL-1β and IL-2 was also of high value in predicting the occurrence of white spot lesions.

**Conclusion:**

After invisible orthodontic treatment, the oral microenvironment, including inflammatory cytokine levels, in adolescent patients changes; in particular, the levels of inflammatory cytokines such as CXCLs and ILs change. CXCL8 expression is significantly associated with the occurrence of white spot lesions and is an important potential target for the prevention and treatment of white spot lesions in the future.

## Background

According to traditional research results, fixed orthodontic appliances are not conducive to the maintenance of oral health, resulting in an increase in local dental plaque, which is the main cause of postorthodontic white spot lesions [1]. In recent years, it has also been reported that the occurrence of white spot lesions in adults or adolescents after orthodontic treatment is related to the number and type of oral cariogenic bacteria and the susceptibility of patients [2]. In the group of adolescent patients who use fixed orthodontic devices, acid production by dental plaque due to a change in oral flora leads to a change in the oral microenvironment and subsequently induces an autoimmune response to control the inflammatory oral environment [3]. Some studies have shown that the expression of proinflammatory cytokines such as interleukins, chemokines and tumor stimulating factors is increased in the saliva of adult patients with white spot lesions after clear aligner orthodontic treatment [4]. The changes in oral inflammatory factor levels in patients with white spot lesions after orthodontic treatment is worthy of attention.

An increasing number of adults and adolescents choose to wear invisible orthodontic appliances, and some studies have reported that invisible orthodontic appliances do not promote the attachment of dental plaque because they are removable. However, our previous study showed that the incidence of white spot lesions in adolescents wearing invisible orthodontic appliances was not lower than that in adolescents wearing fixed orthodontic appliances [5]. The above result may be related not only to the adolescent mental state, hormone levels, whether the patient wears braces, brace cleaning and other clinical factors but also to changes in the oral salivary inflammatory microenvironment, which may also be important influencing factors [6]. In this context, this study aims to further explore the effect of inflammatory factors on white spot lesions and the correlations of inflammatory factors with lesion occurrence by comparing the changes in the inflammatory factor expression profile of the saliva between adolescent patients with and without white spot lesions after clear aligner orthodontic treatment.

## Methods

### Study population and clinical data

In this retrospective clinical study, the sample-size calculation was based on the following formula:

$$N={\left(\frac{{Z}_{1-\alpha /2}\times \sqrt{p\times (1-p)}}{\delta }\right)}^{2}$$

When *α* = 0.05, *p* = 0.8, *δ* = 0.1, Z = 1.96, after calculating, the N = 62. Considering 10% lost to follow-up rate, at least 68 participants were needed. The study protocol was approved by the ethical committee of the School of Stomatology, Air Force Medical University, Xi’an, China. Informed consent forms were signed by all patients or their legal representatives after the protocol and study objective were explained. The potential participants were patients examined during 1 year (2020.03-2021.05) (n = 213) who routinely presented to the Department of Orthodontics. Of these patients, 68 adolescent patients who met the following inclusion criteria as follows were selected: ① adolescents in the permanent dentition period, who aged from 10 to 19 years old according to the WHO standards; ② patients who received clear aligner orthodontic treatment for more than 12 months; ③ patients who had not taken antibiotics or drugs that may cause gingival hyperplasia within the previous 2 months; ④ patients who did not receive cytokine treatment before clear aligner orthodontic treatment; ⑤ patients whose routine blood tests and blood coagulation tests before orthodontic treatment were normal; ⑥ patients who had no infections caused by HBV, HCV, HIV, or CMV, no fever and no bacterial infections within the previous 2 months; ⑦ patients no tumors of any type; and ⑧ patients whose treatment was not complicated by serious disease of the heart, brain, lungs, liver, kidneys or other organs.

The clinical data collected from the patients included age, sex, frequency of brushing teeth per day, duration of tooth brushing, type of toothbrush, type of toothpaste, frequency of mouthwash use, presence of dental caries before treatment, frequency of oral cleaning, frequency of drinking carbonated drinks, frequency of eating with the invisible orthodontic appliance (personal behavior), duration of eating with the invisible orthodontic appliance (required by the doctor) and frequency of cleaning the invisible orthodontic appliance after eating.

### Grouping

After 6 months of treatment, the patients were divided into the following two groups.

For patients in the white spot lesion group (WLG), ① at least one tooth had white spot lesions; ② visible white spots that worsened in a side-by-side comparison of photos taken before and after treatment were regarded as white spot lesions, and other white spots were regarded as developmental white spots; ③ new white spots that appeared during orthodontic treatment were regarded as white spot lesions; and ④ white spot lesions were only confirmed by consensus after visual evaluation by three investigators. The non-white spot lesion group (nWLG) included the remaining adolescent patients. All patients were followed up and evaluated in our hospital after 6 months of treatment.

### Saliva sampling

Three saliva samples were collected from each participant at three different time points:

T0: Before orthodontic appliance placement.

T1: 6 months after orthodontic appliance placement.

The samples were collected in the dental chair between 9 AM and 12 PM, two hours after any food intake. The participants were asked to rinse with water for 30 s and chew a piece of paraffin for 1 min to stimulate salivary flow. Then, saliva was collected in sterilized and preloaded tubes, and a label marked with the patient’s name and collection time was placed on the tubes. All samples were stored at -80 °C for subsequent cytokine detection.

### Metatranscriptome analysis of saliva samples

#### Total RNA collection and mRNA enrichment

Frozen saliva samples were thawed on ice and centrifuged to collect debris. A Lysing Matrix B tube (MP Biomedicals) was used to lyse the debris (twice for 10 min and 30 s). Total RNA was extracted using a MasterPure Complete DNA and RNA Purification Kit (Epientre, Chicago, IL, USA), and DNase was used to eliminate DNA contamination. RNA quality, quantity and integrity were evaluated using a 2100 Bioanalyzer (Agilent Technologies, Santa Clara, CA, USA). Structural oligo (dT) magnetic beads were used to enrich the mRNA, and the enriched mRNA was then randomly interrupted by divalent cations in NEB Fragmentation Buffer (NEBNext ®Ultra RNA Library Prep Kit for Illumina ®).

### cDNA library preparation

cDNA library preparation was performed according to the TruSeq Stranded mRNA protocol (Illumina, San Diego, CA, USA). The first strand of cDNA was synthesized in an M-MuLV reverse transcriptase system with fragmented mRNA as a template and random oligonucleotides as primers. Then, the RNA strand was degraded by RNaseH, and the second strand of the cDNA was synthesized from dNTPs in a DNA polymerase I system. The purified double-stranded cDNA was repaired by terminal repair, subjected to tail addition and sequenced. cDNA of approximately 250–300 bp was screened with AMPure XP beads, the PCR product was amplified by PCR, and the PCR product was purified with AMPure XP beads. After the construction of the library, the library was initially quantified with a Qubit2.0 Fluorometer and diluted to 1.5 ng/µl, and then the insert size of the library was detected with an Agilent 2100 bioanalyzer. After the insert was shown to be of the expected size, qRT‒PCR was performed to quantify the effective concentration of the library accurately (the effective concentration of the library was higher than 2 nM) to ensure the quality of the library.

### Illumina sequencing

After passing the library quality control step, different libraries were sequenced on the Illumina platform after pooling according to the effective concentration and the target off-machine data demand. The basic principle used for sequencing was sequencing while synthesizing (Sequencing by Synthesis). Four kinds of fluorescently labeled dNTPs, DNA polymerase and splice primers were added to the sequencing flow cell for amplification. When each sequenced cluster extends its complementary chain, each fluorescently labeled dNTP can release the corresponding fluorescence. The sequencer captures the fluorescence signal and converts the optical signal into a sequencing peak with computer software; thus, the sequence information of the fragment to be tested is obtained.

### Cytokine measurement

Salivary cytokines were selected based on the results of metatranscriptome analysis; we analyzed all potentially relevant cytokines to obtain a spectrum of cytokines that was as comprehensive as possible. Batch analyses of the salivary samples were performed using a Luminex 200 with a Luminex Kit (R&D Systems). The following cytokines were measured: CXCL1, CXCL2, CXCL8, CCL3, CCL4, interleukin (IL)-1β and IL-2. The samples were thawed, centrifuged, and stained according to the manufacturers’ instructions and the protocol for cell culture supernatants. The concentrations of the cytokines were calculated using a log regression standard curve. If the concentrations were below the quantification limit, they were set to half the detection limit (DL).

### Statistical analysis

Statistical analysis was performed using SPSS24.0 and GraphPad Prism 8.0. Analysis of variance was verified by the Fisher test. For quantitative variables, the independent sample t test was used for intragroup and intergroup comparisons. The cytokines related to white spot lesions were screened by Spearman correlation analysis. A logistic regression equation was used to analyze the correlation between cytokines and white spot lesions. We evaluated the predictive efficacy of single and combined application of the cytokines for the occurrence of white spot lesions with ROC curves. The confidence interval was set to 95%, and differences were considered significant if *P* was < 0.05.

## Results

### Study population and clinical data

We compared average age, sex, body mass index (BMI) and other basic clinical data between the two groups. The differences were not statistically significant, and the distributions of the two groups were comparable (*P* > 0.05) (Table 1).

**Table 1:** Comparison of basic characteristics between patients in the WLG and nWLG

Basic characteristic	WLG	nWLG	*t*/*χ*^2^ value	*P* value
Number	28	40		
Age(y)	14.17 ± 2.09	14.25 ± 1.55	0.162	0.872
Gender				
Male	15	23	0.103	0.748
Female	13	17		
BMI (kg/m^2^)	22.61 ± 1.65	22.71 ± 1.48	0.262	0.794
Number of times brushing daily	3.29 ± 0.53	3.23 ± 0.48	0.490	0.626
Time of brushing each time (min)	6.21 ± 0.88	6.35 ± 1.12	0.536	0.594
Time of appliance wearing per day (h)	19.82 ± 1.32	19.73 ± 1.53	0.132	0.878

### Transcriptome analysis of saliva before treatment

**Fig. 1:**
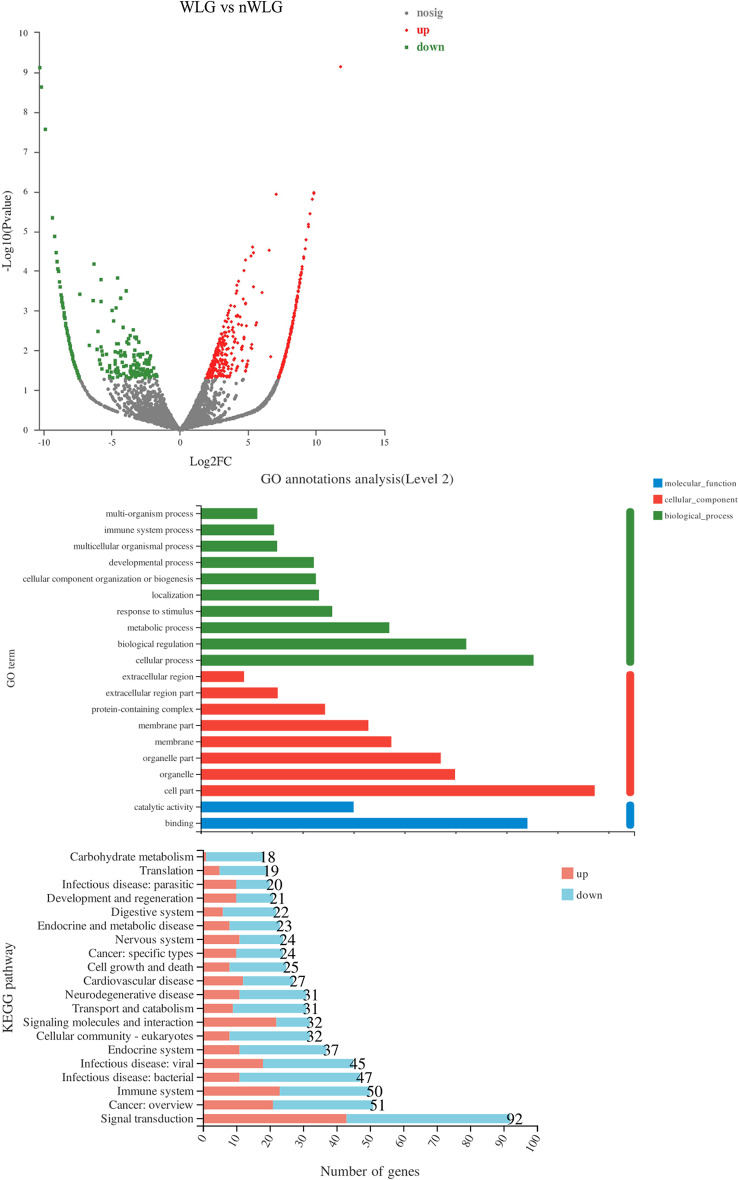
Analysis of differentially expressed genes and pathways in the saliva of patients before treatment

We randomly selected 7 patient saliva samples from each group for transcriptome analysis and found that there were differences in the expression levels of 995 genes in saliva between the two groups, with 419 genes with decreased levels and 576 genes with increased levels in the WLG group. Further GO and KEGG pathway analysis showed that there were differences in the transcription levels of many inflammation-related cytokines, such as CXCL1, CXCL2, CXCL8, CCL3, CCL4, IL-1 β and IL-2, between the groups (Fig. 1).

### Expression levels of inflammation-related cytokines before and after treatment

There were no significant differences in the levels of CXCL1, CXCL2, CXCL8, CCL3, CCL4, IL-1 β and IL-2 in saliva between the two groups before treatment (*P* > 0.05). After treatment, the levels of CXCL8, CCL3, CCL4, IL-1 β and IL-2 in saliva were significantly increased in both groups, and the levels of inflammation-related cytokines in the WLG group were significantly higher than those in the nWLG group (*P* < 0.05) (Table 2).

**Table 2:** Comparison of salivary cytokines between T0 and T1

Cytokines	Time	WLG (N = 28)	nWLG (N = 40)
CXCL1(ng/mL)	T0	12.46 ± 2.70	12.21 ± 2.19
	T1	12.60 ± 1.90	12.09 ± 2.33
CXCL2(ng/mL)	T0	10.33 ± 2.13	10.90 ± 2.56
	T1	10.42 ± 2.43	10.68 ± 2.33
CXCL8(ng/mL)	T0	18.13 ± 2.99	18.81 ± 3.35
	T1	25.09 ± 2.45^#*^	22.48 ± 3.37^*^
CCL3(ng/mL)	T0	5.02 ± 1.08	5.37 ± 1.22
	T1	8.09 ± 2.16^#*^	6.98 ± 1.78^*^
CCL4(ng/mL)	T0	1.94 ± 0.46	1.85 ± 0.48
	T1	3.60 ± 0.99^#*^	3.11 ± 0.85^*^
IL-1β(ng/mL)	T0	7.93 ± 1.78	8.58 ± 2.01
	T1	11.23 ± 2.82^#*^	9.65 ± 2.31^*^
IL-2(ng/mL)	T0	3.75 ± 0.39	3.79 ± 0.36
	T1	6.10 ± 1.67^#*^	4.89 ± 1.59^*^

^#^: compared with the nWLG group, P < 0.05;^*^ compared with before treatment, P < 0.05

### Analysis of the relationship between inflammation-related cytokines and white spot lesions

Spearman correlation analysis showed that the occurrence of white spot lesions after invisible orthodontic treatment was positively correlated with salivary CXCL8, CCL3, CCL4, IL-1 β and IL-2 levels (*P* < 0.05) but not with salivary CXCL1 and CXCL2 levels (*P* > 0.05) (Table 3).

**Table 3:** Analysis of the relationship between inflammation-related cytokines and white spot lesions

Cytokines	Spearman r	P
CXCL1	0.091	0.463
CXCL2	-0.091	0.459
CXCL8	0.411	< 0.001
CCL3	0.241	0.048
CCL4	0.264	0.030
IL-1β	0.279	0.021
IL-2	0.346	0.004

### Logistic regression analysis to screen for risk factors for white spot lesions

Logistic regression analysis showed that the elevated levels of CXCL8, IL-1 β and IL-2 were independent risk factors for the occurrence of white spot lesions in patients after invisible orthodontic treatment (*P* < 0.05) (Table 4).

**Table 4:** Binary logistic regression analysis results

Index	*β*	*S. E*	*Wald*	*P*	*OR*	95% C.I.	
						lower	upper
CXCL8	0.297	0.120	6.065	0.014	1.345	1.062	1.703
CCL3	0.150	0.160	0.880	0.348	1.161	0.850	1.588
CCL4	0.259	0.353	0.537	0.464	1.295	0.649	2.586
IL-1β	0.326	0.140	5.456	0.020	1.386	1.054	1.822
IL-2	0.449	0.201	5.010	0.025	1.567	1.057	2.323

### Evaluation of the predictive value of risk factors for the occurrence of white spot lesions in patients

**Fig. 2:**
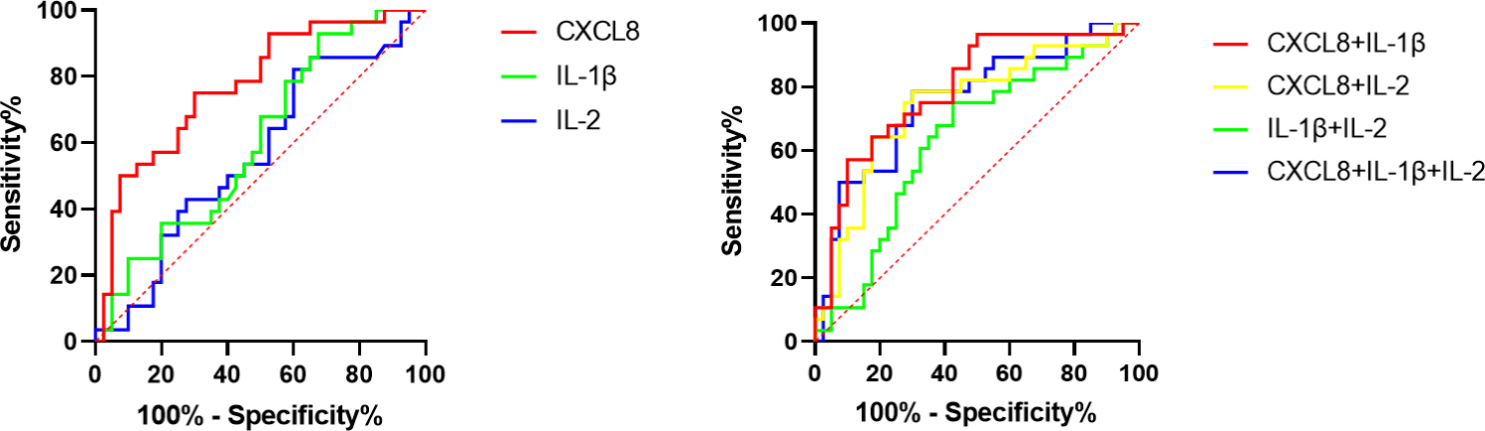
Predictive efficacy of each cytokine when applied alone or in combination for the occurrence of white spot lesions

ROC analysis showed that CXCL8 had a high independent predictive value for white spot lesions after invisible orthodontic correction in adolescents, with an area under curve (AUC) of 0.773. Although IL-1β and IL-2 had little independent predictive value for the occurrence of white spot lesions, they had higher predictive value in combination with CXCL8, with AUCs of 0.795 and 0.745, respectively (Table 5; Fig. 2).

**Table 5:** Predictive efficacy of each cytokine for the occurrence of white spot lesions

Cytokines	Optimal cut-off point	Sensitivity (%)	Specificity (%)	AUC	95% C.I.	*P* value
CXCL8	< 23.44 ng/mL	75.00	70.00	0.773	0.660 ~ 0.886	< 0.001
IL-1β	< 12.36 ng/mL	92.86	32.50	0.606	0.472 ~ 0.740	0.140
IL-2	< 6.00 ng/mL	82.14	40.00	0.564	0.425 ~ 0.703	0.373
CXCL8 + IL-1β	/	57.14	90.00	0.795	0.686 ~ 0.904	< 0.001
CXCL8 + IL-2	/	78.57	70.00	0.745	0.622 ~ 0.867	< 0.001
IL-1β + IL-2	/	7500	57.50	0.638	0.503 ~ 0.772	0.055
CXCL8 + IL-1β + IL-2	/	78.57	70.00	0.760	0.642 ~ 0.878	< 0.001

## Discussion

With an increasing number of patients choosing nonbracketed invisible orthodontic treatment, patients and doctors should pay more attention to the prevention and treatment of complications and the potential risks after treatment [7]. During clinical treatment, we found that in the invisible orthodontic group, many adolescent patients had white spot lesions after wearing the invisible orthodontic appliance, and the prevalence of this phenomenon in this group may not be lower than that in fixed orthodontic patients [8]. The etiology may be related to the ability of the invisible orthodontic appliance to disrupt homeostasis of the oral environment in adolescent patients and induce a series of inflammatory responses caused by changes in the microflora; the inflammatory factors involved in this process are the focus of this study [9, 10]. Homeostasis of the oral environment plays an important role in inhibiting the growth of oral cariogenic bacteria. Damage to the oral mucosa, disorder of the flora colonizing the oral cavity, induction of an oral inflammatory response and disruption of the oral immune balance may lead to disruption of oral homeostasis and increase the possibility of invasion of cariogenic bacteria [11, 12]. Different oral bacteria can induce different cytokine expression profiles due to their different cariogenic abilities. It is generally believed that some important cariogenic bacteria and opportunistic cariogenic bacteria can cause a strong inflammatory response [13]. For example, actinomycetes can reduce the expression of IL-1β and increase the expression of IL-6 and CXCL8 in oral mucosal epithelial cells [14].

Previous studies have investigated people with white spot lesions after fixed orthodontic treatment; in addition to an increase in the dental plaque index, which is an important factor affecting white spot lesions, the type and number of cariogenic bacteria in saliva also increased compared with those of individuals without treatment, the expression levels of some inflammatory factors increased, and the incidence of enamel demineralization and white spot lesions increased [15]. Orthodontic treatment is a long-term process during which oral mucosal epithelial cells may secrete inflammation-related cytokines and release them into the gingival crevicular fluid and saliva under the stimulation of orthodontic appliances [16]. However, research on the relationship between oral inflammatory cytokine levels and white spot lesions is still limited. Within 1 month after the initiation of orthodontic treatment, the level of oral inflammation in adult patients who received fixed orthodontic treatment or clear aligner orthodontic treatment was significantly higher than that in adults without treatment, while the expression levels of ILs, TNF-α and other inflammatory factors in the gingival crevicular fluid and saliva of adult patients who received clear aligner orthodontic treatment were lower than those who received fixed orthodontic treatment in the same period [17]. This shows that although all orthodontic treatments can disrupt local oral homeostasis due to physical stimulation, inflammatory factors play an important role in this process. Adolescent patients experience significant changes in sex hormone levels, which can lead to the induction of gingivitis and other oral inflammatory diseases. In addition, compared with adult patients, adolescents have a shorter permanent tooth eruption time, a lower degree of enamel mineralization, and a higher likelihood of experiencing demineralization [18]. Therefore, it is of great clinical significance to take adolescent patients as the research object and explore which cytokines in saliva are related to the occurrence of white spot lesions and whether these factors have predictive value for the occurrence of white spot lesions.

A review of previous studies and our previous results showed that in young patients who wear removable invisible orthodontic appliances for a long time, the oral cavity is in a state of chronic inflammation due to changes in hormone levels in the body and the failure of individual patients to clean their teeth or appliances after long-term use [19]. Members of the CXC chemokine subfamily and interleukins may play important roles in this process. In this study, we first found that there were differences in the transcription levels of inflammatory cytokines such as CXCL1, CXCL2, CXCL8, CCL3, CCL4, IL-1 β and IL-2 in saliva between the WLG and nWLG. Verifying the transcriptome results, the expression levels of CXCL1, CXCL2, CXCL8, CCL3, CCL4, IL-1 β and IL-2 in the saliva of WLG patients were significantly higher than those in the saliva of nWLG patients. Previous studies have shown that CXCL1, CXCL2 and other genes are related to the production of oral streptococcal biofilms, which may affect local infiltration of inflammatory immune cells into the oral cavity by stimulating the production of other inflammatory cytokines, such as TNF, or the activation of immune signaling pathways [20]. CXCL8 is mainly produced by monocytes-macrophages and can also be secreted by epithelial cells and endothelial cells in response to some stimuli. It is a strong leukocyte chemoattractant and neutrophil activator. Previous studies have shown that increasing the expression level of CXCL8 can counteract the inhibitory effect of oral bacteria on leukocytes to some extent in patients with white spot lesions, but an expression level that is too high can cause a local inflammatory cascade in the oral cavity and form a microenvironment conducive to the growth of oral flora [21]. CCL3 and CCL4 are considered potential biomarkers of oral squamous cell carcinoma, and they are also related to monocyte aggregation and inflammatory infiltration, but there are few studies on their relationship with oral flora [22]. IL-1 β and IL-2, which are common proinflammatory cytokines in the interleukin family, can participate in the colonization and proliferation of *Campylobacter conjunctiva* and *Candida albicans* and are important factors leading to increased local oral inflammation [23, 24].

Considering our preliminary screening data and previous studies, the above inflammatory cytokines may be associated with white spot lesions. Through correlation analysis and logistic regression analysis, we found that CXCL8, CCL3, CCL4, IL-1 β and IL-2 levels were significantly correlated with the occurrence of white spot lesions, while only CXCL8, IL-1 β and IL-2 may be independent risk factors for white spot lesions. After evaluating the predictive value of independent risk factors for the occurrence of white spot lesions, we found through ROC curve analysis that CXCL8 was the most effective in independently predicting the occurrence of white spot lesions. Although the independent predictive value of IL-1β and IL-2 was poor, they had clear predictive value when combined with CXCL8. With regard to the relationship between CXCL8 and the occurrence of white spot lesions, previous studies have shown that resident oral microflora, such as oral streptococcus, have an effect on the level of CXCL8 secreted by oral epithelial cells and are also sensitive to changes in CXCL8 levels [21]. In addition, the proportion of oral microflora, such as oral streptococcus, was also related to the change in CXCL8 levels in gingival crevicular fluid in patients with gingivitis and healthy people. In addition, some studies have shown that the stability of oral mucosal cells is related to the level of CXCL8 through the spatial transcriptome, and CXCL8 may change the local inflammation level and internal environment of the oral cavity by recruiting lymphocytes and indirectly affect the distribution and types of oral flora. These results strongly suggest that CXCL8, IL-1 β and IL-2 may be involved in the occurrence of white spot lesions after clear aligner orthodontic treatment and are all independent risk factors, with CXCL8 playing an important role in white spot lesions and oral flora imbalance. It is speculated that wearing an invisible orthodontic appliance for a long time may reduce the self-cleaning ability of the tooth surface, limit the washing and cleaning effect of the saliva and tongue on the tooth surface, and generate conditions suitable for the reproduction of pathogenic bacteria, thus destroying the microecological balance of the oral flora and affecting biofilm attachment. Previous studies have shown that long-term wearing of invisible orthodontic appliances can increase the abundance of actinomycetes and Rosella in supragingival plaque [5]. A previous study by our group also showed that the abundance of Actinobacillus, Rosella and anaerobes increased significantly in the saliva of adolescent patients who wore invisible orthodontic appliances and developed white spot lesions. Among these flora, anaerobes have an obvious activating effect on monocytes-macrophages, and abnormally activated monocytes-macrophages may cause changes in the local immune and inflammatory microenvironment via overexpression of inflammatory cytokines such as CXCL8, which further aggravate disorder of the oral microecology and eventually lead to an increase in the incidence of white spot lesions [5]. In addition, the thickening and increased distribution of plaque biofilms caused by invisible orthodontic appliances also provide an environment suitable for the occurrence of oral infectious diseases and pathogen invasion. *Streptococcus mutans*, staphylococci and Lactobacillus can activate local immune cells and release inflammatory mediators such as TNF-α and CXCL8; this may also be the potential mechanism of white spot lesion development related to the increase in CXCL8 levels. However, regardless of the possible mechanism, the role of CXCL8 in the occurrence and development of white spot lesions and the relationship of CXCL8 with the oral microenvironment need to be further explored [25, 26].

Although we have observed many changes in inflammatory cytokine levels and proved the correlation between the expression levels of inflammatory cytokines and the incidence of white spot lesions in adolescent patients after invisible orthodontic correction, there is still a lack of research on the relationship between inflammatory cytokines and oral cariogenic bacteria. At the same time, due to the patient’s personal reasons, COVID-19 epidemic reasons, etc., the follow-up rate at 12 months after treatment is low, so long-term follow-up results are lacking. In future studies, we will continue to follow up the enrolled patients to collect the incidence of WSL and the changes of oral inflammation microenvironment after long-term treatment (more than 12 months). In the meantime, we will focus on CXCL8 by using metabonomics analysis, flora identification, combinomics analysis and other methods to explore its correlations with oral flora types, quantitative flora changes and flora imbalance and further clarify the relationships among inflammation, the oral flora and white spot lesions to provide a theoretical basis for the prevention and treatment of white spot lesions after invisible orthodontic treatment.

## Conclusion

This study found that after invisible orthodontic treatment, the oral microenvironment, including inflammatory cytokine levels, in adolescent patients changes; in particular, the levels of inflammatory cytokines such as CXCL and IL change. CXCL8 expression is significantly associated with the occurrence of white spot lesions and is an important potential target for the prevention and treatment of white spot lesions in the future.

## Data Availability

All data generated or analysed during this study are included in this published article.

## Abbreviations

AUC: Area under curve
BMI: Body mass index
IL: Interleukin
nWLG: Non-white spot lesion group
ROC: Receiver operating characteristic
WLG: White spot lesion group
